# Medicine

**Published:** 1906-06

**Authors:** George Parker


### Progress of the Medical Sciences.

### MEDICINE.

**The treatment of internal hemorrhage** is a subject which has been involved in much uncertainty. The majority of these attacks of bleeding come to a speedy end of themselves, and it is hard to say when we have given a hæmostatic whether the ending is due to the drug or to nature. To increase the confusion, some drugs have been recommended which act as vaso-contractors and others as dilators, and others again which can hardly be absorbed at all. Thus, as W. E. Dixon[1] points out, if we give the tannins, only one per cent. is absorbed, and that in the almost inert form of gallate of sodium, which can neither cause clotting nor constrict the vessels. In the same way adrenalin, unless injected intravenously or applied direct to a bleeding point, produces no effect on the vessels. Certain facts, however, have been ascertained as to the action of the chief vaso-contractors. The constriction they cause is most marked in the vessels of the splanchnic area, less marked in those of the skeletal muscles, and far weaker in the vessels of the lungs, the brain, the liver, and the coronary vessels. Therefore the rise of pressure produced in the first and second groups quite overpowers the slight contraction caused in the vessels of the lungs, the liver, the brain, and the heart. These organs actually

[1] *Lancet*, 1906, i. 825.

become congested and their vessels dilated under the action of general vaso-contractors. Dixon has shown that these results hold good for all the ordinary drugs, and it is therefore clear that not only we cannot rely upon them for checking hemorrhage from the lungs, the brain, or the liver, but they are actually injurious. These facts as to the relative contraction of the vessels in different organs are based on the results obtained by the perfusion of blood through the isolated organs, and the drugs employed comprise adrenalin, ergot, digitalis and its allies, and the group containing veratrine, barium, and lead. Some of them act on the muscle directly and some through the nervous system, but the final result is the same for them all. It will be seen that hemorrhages from the uterus, the intestines, and other parts of the splanchnic area, are undoubtedly affected by these drugs, and that the common gynæcological use of ergot is rather supported by these experiments. For gastric bleeding again their use is considerable, if the injured vessel is of a size which can be efficiently constricted. Adrenalin requires to be injected into the veins, as when it is given by the mouth or subcutaneously it appears to have no effect on the vessels, though it is just possible that superficial gastric vessels might be constricted by the drug when swallowed. W. P. Herringham has no doubt of its value in gastric hemorrhage, and advises its regular employment.[1] Hemorrhage from dilated œsophageal veins due to cirrhosis of the liver will clearly not be aided by any vaso-constrictors.

For hæmoptysis we must clearly turn to some other remedies. First we may consider the vaso-dilators. If they dilate the vessels of the systemic circulation and fill the splanchnic area they must tend to deplete the lungs as Foxwell pointed out,[2] and so far hinder hæmoptysis. We cannot expect them to stop hemorrhage of every type. In ordinary cases of hæmoptysis such as we meet with in phthisis Francis Hare[3] reported marked success from inhalations of nitrite of amyl. Thus in twelve cases the bleeding ceased within three minutes, and the immediate relief which the patients experienced had also a great effect in allaying the mental excitement which accompanies such attacks. It is certainly a remarkable thing that the evanescent action of the drug upon blood pressure should suffice for the formation of clot, but confirmatory accounts have been given by other observers. E. T. Smith[4] reported: "As soon as the nitrite was given the next clearing of the air passages produced blood-stained mucus only. The next cough showed only mucus." The case was apparently one of rupture of an atheromatous vessel in a patient suffering from interstitial nephritis.

Other remedies which act in much the same way as the vaso-dilators are saline purgatives and morphia. The latter,

1 *Clin. J.*, 1906, xxvii. 337. 2 *Essays on Heart and Lung Disease*, 1895.
3 *Lancet*, 1904, ii. 522, 942. 4 *Brit. M. J.*, 1906, i. 917.

though it has no direct action on the heart or vessels, diminishes all sensory reflexes and excitement which would "induce cardiac acceleration and reflex rise of blood pressure." The value of these two remedies is incontestable unless the bleeding has been extreme.

When we come to the arrest of cerebral hemorrhage the problem at first sight seems to be precisely like that of the treatment of hæmoptysis. Vaso-constrictors act on the general circulation and increase the cerebral congestion, while their effect on the torn vessel is little or none. Hence we may gather some indication for vaso-dilators and purgatives, but serious doubt has been thrown on the advisability of this treatment since it has been shown that with large intra-cranial hemorrhages anæmia of the medulla is produced, and that a protective rise of tension to an extraordinary degree is brought about in order to keep the medulla supplied with blood. Hence American writers like Cook and Lashing[1] lay stress on the danger of reducing the tension in cerebral apoplexy and advocate surgical removal of the clot. Probably when there is but a slight rise of pressure indicating a local bleeding alone, and provided we are otherwise certain that we are dealing with a hemorrhage and not a vascular occlusion, we should be justified in using vaso-dilators or purgatives. If the pulse pressure is extremely high those remedies will only hasten the end.

Now besides those agents which act on the vaso-motor mechanism there are yet other means of arresting hemorrhage. There are the drugs which increase the clotting power of the blood, gelatine, the calcium salts, and possibly the iodides. The use of gelatine has two dangers, one is that impure gelatin containing tetanus germs may be used, and the other is that the treatment may be carried too far and thrombosis and embolism produced. To meet the first danger sterilisation of gelatin in steam under pressure at 115° for thirty minutes should be carried out before subcutaneous injections. Lancereaux[2] recommended that 7 ounces of a 2½ per cent. solution of gelatine in normal saline fluid should be injected every third or fourth day in aneurysms, but rectal injections of half a pint of a 5 per cent. solution three times a day for several days may be given for most internal hemorrhages, and satisfactory results have been obtained on the whole. Moreover in these rectal injections the dangers just referred to seem to be reduced to a minimum.

Wright and Paramore[3] have reinvestigated the blood-clotting power of calcium salts. The maximum effect appears to be obtained within 45 minutes, and lasts for several days. The quantity recommended is 60 grains of calcium chloride or lactate by the mouth in a single dose. The latter salt is more pleasant to the taste, and may be given by subcutaneous injection in 5 to 10 grain doses dissolved in thirty times its

[1] *Johns Hopkins Hosp. Rep.*
[2] *Edin. M. J.*, 1904, N.S., xvi. 359. [3] *Lancet*, 1905, ii. 1096.

weight of water. Some patients do not readily absorb calcium salts from the stomach, and for them hypodermic injections of the lactate seem to be preferable. A curious fact was also noted that magnesium carbonate is a powerful coagulating agent similar to calcium in its effects, which explains its use in urticaria and other serous exudations. To maintain permanently high coagulation it is necessary to test the blood frequently and stop the drug when the maximum effect is reached, but for the temporary arrest of hemorrhage this is not required.

**The Adams-Stokes affection of the heart** has long been difficult of explanation. The extraordinary slowing of the pulse sometimes to ten or twelve beats in the minute as in a case formerly reported by me in this *Journal*, and the apoplectiform attacks from time to time with few signs of cardiac disease either during life or *post-mortem* point to some unknown action of the cardiac mechanism. It has been also noticed that numerous jugular pulsations co-exist with the infrequent radial ones, and by a method of simultaneous tracings perfected by John Hay it was found that in these and similar affections the interval between the auricular and ventricular pulsations is abnormally long. Thus there is a slowing of the conduction of the wave of contraction. Now His had shown that the only connection between auricle and ventricle is a small bundle of fibres in the auriculo-ventricular septum. He suggested in 1904 that a lesion in this bundle might produce the symptoms of the Adams-Stokes type by causing a block in the course of the wave. The experiments of Stannius in 1856 had shown that compression of this septum results in a different rate of contraction for the auricle and ventricle. His thought that it is interference with this bundle of fibres which by blocking conduction sets free the ventricle to beat at its own inherent slow rate. Erlanger[1] devised a clamp to compress this bundle alone, and found that he could produce a ventricular rhythm only half or a quarter as rapid as that of the auricle, and finally that the ventricle could be stopped altogether while the auricle went on. Moreover it was shown that neither changes in the vagus, the sympathetic or the heart muscle could produce these symptoms. It was seen that this condition of heart block occurs in many other conditions besides the Adams-Stokes disease, and that the absence of macroscopic findings in the latter may be due to the smallness of this bundle. Finally Stengel[2] met with an actual case with typical slow pulse, seizures, and rapid pulsation of veins. Careful dissection showed that the bundle of His and that alone was involved by a patch of sclerotic endocarditis. The coronary arteries were neither calcareous or narrowed, which disposes of Greiwe's

[1] *J. Experiment Med.*, 1906, viii. 8. [2] *Am. J. M. Sc.*, 1905, cxxx. 1083.

theory as far as this disease is concerned, that coronary changes produce bradycardia by damaging the muscle and so causing an increase in the refractory period. In Stengel's patient the radial pulse was at times only 16 or 20 per minute, while the venous pulsation was 80 to 100. Before and during the syncopal seizures the radial pulse would cease from 20 to 130 seconds though the venous pulsation went on the whole time, showing that absolute heart block occurred in those attacks. G. A. Gibson,[1] like Hay and Mackenzie, accepts this "block" theory as explaining cases of bradycardia where the jugular pulsations exceed the radial ones, while in cases where the heart is slowed down as a whole there is, he thinks, deficient stimulation or lessened excitability of the heart muscle. These are two instances of irregular pulsation of every part of the heart, where, without loss of conductivity, the ventricular systole, like the auricular, may often be abortive. As Erlanger pointed out, the auricular systole can be directly influenced by atropine, while the ventricular cannot. Thus if a block in conduction exists atropine is practically useless. Hay[2] reported a case like Stengel's with an auricular rate double that of the ventricle, and here too marked pathological changes were found in the septum.

**Divers' Paralysis.**—Leonard Hill has shown that under abnormal pressure large quantities of nitrogen are dissolved in the blood, and if the pressure is rapidly lowered this gas is set free in it and may pass into the peri-vascular spaces.[3] It is probable that paralysis is due to the setting free of this gas in the vessels of the spinal cord when the pressure is reduced too quickly. A patient of Hale White suffered from three separate attacks after working at depths of 130, 150, and 162 feet, another man reached the depth of 189 feet, and Greenwood,[4] who has investigated the matter afresh, mentions a descent to 204 feet, which was equal to $+ 88\frac{1}{2}$ lb. air pressure. He obtained a compression chamber, and was himself subjected to a pressure of $+ 92$ lb., which corresponds with a depth of water of 210 feet, or seven atmospheres. No ill results followed, as he had the reduction done so slowly as to take twenty minutes for each atmosphere, and during compression moved every part of his body so as to maintain the capillary circulation. Subsequent experiments showed the harmlessness of these great pressures provided these two rules are observed. Some curious results as to $CO_2$ absorption were obtained. It was found that when large quantities of $CO_2$ were allowed to be present in the compressed air, equal to 1.5 or 2 per cent. under normal pressure, no interference with respiration took place. Haldane and Priestley's law that the percentage of $CO_2$ in the alveoli of the lungs is

[1] *Edinb. M. J.*, 1905, N.S., xviii. 9. [2] *Brit. M. J.*, 1905, ii. 1072.

[3] *Lancet*, 1905, ii. 1. [4] *Brit. M. J.*, 1906, i. 912.

always the same in health, or about 5 per cent., was also shown to be true under altered pressures. As the pressure rises the percentage of $CO_2$ present in the alveoli varies inversely with it, so that the quantity of the gas remains constant.

GEORGE PARKER.

### SURGERY.

